# The Role of the Gut Microbiome and Microbial Dysbiosis in Common Skin Diseases

**DOI:** 10.3390/ijms25041984

**Published:** 2024-02-06

**Authors:** Izabella Ryguła, Wojciech Pikiewicz, Beniamin Oskar Grabarek, Michał Wójcik, Konrad Kaminiów

**Affiliations:** 1 Faculty of Medical Sciences in Katowice, Medical University of Silesia, 40-752 Katowice, Poland; izabella.ryg@gmail.com; 2 Collegium Medicum—Faculty of Medicine, WSB University, 41-300 Dabrowa Gornicza, Poland; wojciech.pikiewicz@wsb.edu.pl (W.P.); beniamin.grabarek@wsb.edu.pl (B.O.G.); lekmichalwojcik@gmail.com (M.W.)

**Keywords:** microbiome, gut, dermatologic conditions, acne, psoriasis, alopecia areata, atopic dermatitis, dermatology, skin

## Abstract

Dermatoses are an increasingly common problem, particularly in developed countries. The causes of this phenomenon include genetic factors and environmental elements. More and more scientific reports suggest that the gut microbiome, more specifically its dysbiosis, also plays an important role in the induction and progression of diseases, including dermatological diseases. The gut microbiome is recognised as the largest endocrine organ, and has a key function in maintaining human homeostasis. In this review, the authors will take a close look at the link between the gut–skin axis and the pathogenesis of dermatoses such as atopic dermatitis, psoriasis, alopecia areata, and acne. The authors will also focus on the role of probiotics in remodelling the microbiome and the alleviation of dermatoses.

## 1. Introduction

Dermatological diseases are an important public health problem. Worldwide, between 30% and 70% of the population suffer from dermatoses, with dermatological conditions being the most common reason for consultation in general practice and the fourth most common cause of non-fatal burden [1]. Dermatoses are highly associated with a negative impact on the quality of daily and working life, are a source of stigma, and can contribute to psychological problems [2].

The tremendous development of technologies related to DNA extraction and 16S ribosomal RNA (rRNA) apposition sequencing analysis has allowed bacterial genes to be explored [3]. Between 75 and 200 trillion bacterial organisms make up the human microbiota. In comparison, the number of human somatic cells ranges from 50 to 100 trillion [4]. In addition to the number, it is also astonishing that the native microbes inhabiting the human body play a significant role in maintaining host homeostasis [5]. A huge contribution of the gut microbiome, or more precisely its dysfunction, has been postulated in many conditions, including dermatological diseases [6,7]. It is important to draw the attention of the public, and especially of clinicians involved in the management of patients, to the enormous role played by certain bacteria in maintaining the health of their host. Therapies aimed at rebalancing the gut microbiota may provide a useful method for the prevention and treatment of skin diseases. 

The aim of this review is to describe the role of the gut microbiome in maintaining human homeostasis and the impact of gut dysbiosis on the development of dermatological conditions such as atopic dermatitis, psoriasis, alopecia areata, and acne. It also focuses on discussing the role of probiotics in alleviating the course of these dermatological diseases.

## 2. Gut Microbiome

To begin with, it is worth focusing on the terms microbiota and microbiome. Sometimes these terms are used interchangeably, but it should be noted that ‘microbiota’ refers to a group of commensal, symbiotic, and pathogenic microorganisms found in an established environment, while ‘microbiome’ is a broader term and encompasses the entire range of microorganisms, including bacteria, viruses and fungi, their genes and metabolites, and the surrounding environment [5].

Human skin provides a barrier against external factors and an abundant ecosystem for numerous microorganisms. The approximate number of organisms residing on the skin is 10^12^. Interestingly, it is not the skin that hosts the highest number of microorganisms. The largest number (10^14^ microorganisms) reside in the intestines, forming the intestinal microbiota. The total weight of the gut microbiota is about 1.5 kg, which is about the weight of the liver [8,9,10,11]. Study results confirm that, in the human gut, the microbiome consists of 3.3 million genes responsible for the production of thousands of metabolites, while there are 150 times fewer genes in the human genome [12,13].

As reported in a study by Noelle Younge et al., humans acquire the microbiota during prenatal development. Oral microbiota and meconium at the time of caesarean section were present not only in neonates born on time, but also in those born prematurely at 24 weeks’ gestation. This study challenged the claim that the foetus develops in a sterile, clean environment [14]. After birth, during the first years of life, the intestine undergoes a gradual colonization so that, around the age of three to five, it reaches, with its bacterial composition, the microflora profile of an adult [15]. According to other sources, the gut microflora profile of an adult is already reached at the age of 2–3 years [3]. For this reason, the first years of life are the most important for the formation of host microflora, which is ultra-sensitive to environmental factors such as antibiotics and breastfeeding [16]. The intestinal microflora of breast-fed infants is rich in species related to human milk oligosaccharide metabolism. Approximately 30% of an infant’s microbiota is derived from breast milk, and a reduction in the diversity of the intestinal microbiota has been reported in infants fed a milk-replacement mixture [17,18]. The dominant bacterial taxa of the gut profile of healthy adults are *Firmicutes*, *Bacteroidetes*, *Actinobacteria*, *Proteobacteria*, *Fusobacteria*, and *Verrucomicrobia.* However, as much as 90% of the microflora consists of *Firmicutes* and *Bacteroidetes*. However, differences in the composition and proportions of the microbiota may still be present from person to person [19,20].

It is worth noting that each human organism acquires its own unique composition of intestinal microflora. Once established, the set of microorganisms comprising the gut microbiome is relatively constant throughout life. However, certain factors can have a negative impact on the profile of the gut microbiome, leading to dysbiosis [21]. By the term ‘dysbiosis’, the authors mean a situation in which there is a loss of beneficial organisms in the microbiota profile, an exaggerated increase in potentially harmful organisms, or a reduction in the overall diversity of the microflora [13]. The composition of the gut microbiota is influenced by the following factors, among others: host genetics, diet, antibiotic use, body mass index, and lifestyle (physical activity, smoking, occupation, sleep, and stress) [22,23,24,25,26,27,28,29]. The gut microbiome is negatively affected by a diet rich in processed foods, fats, and sugars, and low in fibre. This type of diet alters the microbiota profile towards a pro-inflammatory profile abundant in *Proteobacteria* and *Desulfovibrionaceae* [30]. In contrast, it should be added that diets abundant in fruit and vegetables, whole grains, and probiotic foods are characterised by beneficial effects on the diversity of the host microbiota [31,32]. The profile of the gut microflora may also vary depending on the geographical location in which the organism resides [33].

An imbalance in the diversity and profile of the gut microbiota implies microecological dysfunction of the gut, leading to adverse modifications in gut bacterial metabolism and immune responses. These changes affect physiological as well as pathological activities, and are relevant to human life [34].

Under normal conditions, the gut microbiome is responsible for nutrient metabolism, xenobiotic and drug metabolism, natural defence against infection and immunomodulation [35].

Humans lack the ability to digest and derive energy from dietary fibre and resistant starch due to the lack of appropriate enzymes in their bodies. In contrast, gut bacteria derive 10–30% of their energy precisely from fibre [36,37,38]. Some species comprising the gut microbiota are equipped with the ability to produce unique enzymes that lead to the fermentation of these carbohydrates and the formation of gases, organic acids, alcohols, and short-chain fatty acids (SCFAs) from them [39,40]. The SCFAs produced—more specifically, butyrate, propionate and acetate—create an environment in the gut with a relatively low pH, not allowing pathogens to proliferate, and showing antimicrobial activity [34]. SCFAs also exhibit extensive anti-inflammatory properties through contact with immune cells, the release of reactive oxygen species (ROS), and the release of cytokines. It is through this that the microflora can induce or prevent inflammation. Butyrate, for example, inhibits inflammation by suppressing IL-12 production and increasing IL-10 production in monocytes. SCFAs have also been implicated in maintaining the tightness of the intestinal barrier by promoting the production of mucin glycoproteins responsible for creating intestinal integrity [41]. Through the leaky gut pathway, microbial metabolites can enter the bloodstream, resulting in the production of cytokines and inflammatory mediators [42]. In addition, the gut microflora and its metabolites reach the circulation, accumulate in the skin, and can alter skin homeostasis [43]. Overall, the gut microflora induces maturation of the innate and acquired immune system [34].

In addition to SCFAs, the gut microbiome also produces secondary bile acids, cortisol, gamma-aminobutyric acid (GABA), tryptophan, serotonin, and dopamine [44].

Intestinal dysbiosis associated with changes in the abundance or composition of the microbiota results in the pathophysiology of many conditions, such as diabetes and obesity [45], allergies in a broad sense, including food allergies [46], cardiovascular disease [47], inflammatory bowel disease [48], chronic kidney disease [49], mental illness [50], rheumatological arthritis [51] and, the exact focus of this review, dermatological conditions.

All compounds that are formed in the gut can either have a direct effect on skin receptors or interact with commensal bacteria residing in the skin. It should also be added that the gut–skin axis does not operate in a unidirectional mechanism, but in a bidirectional manner. Certain factors acting on the skin contribute to the appearance of the gut microbiota. For example, skin exposure to ultraviolet B (UVB) radiation leads to an increase in serum vitamin D levels, which has a beneficial effect on the gut microbiota, increasing its diversity [52,53].

In this paper, the authors will discuss the role of the gut–skin axis in the pathogenesis of four dermatological conditions: atopic dermatitis, psoriasis, acne, and alopecia areata. The authors will also focus on the role of probiotics in remodelling the microbiome and the alleviation of dermatoses.

## 3. Gut Microbiome and Probiotics in Dermatologic Conditions

### 3.1. Atopic Dermatitis

Atopic dermatitis (AD), also known as atopic eczema or hereditary allergic eczema, is a treatment-resistant, inflammatory, chronic dermatosis with a complex and multifactorial pathogenesis that is characterised by persistent pruritus, extremely dry skin, and erythema [34,54,55]. Pruritus is a major symptom among individuals struggling with AD and is not uncommonly produced through non-histaminergic signalling pathways, resulting in persistence despite medication, significantly reducing quality of life [56,57,58]. An increased risk of anxiety, depression and other mental health conditions has also been confirmed. AD is also associated with a financial burden for patients due to prolonged treatment with numerous medications and dermocosmetics [59]. AD can occur at any age, but most cases have their onset in infancy [60]. In the adult population in developed countries, AD has a prevalence of 10%, and among the paediatric population the prevalence is 20% [55]. In developing countries, the prevalence is lower, but steadily increasing [61].

Factors such as genetics, environment, and immunity contribute to AD [62]. AD patients have an imbalance between Th1 and Th2 cells, with Th2 dominance. Associated with Th2 are the cytokines IL-4, IL-5, IL13, and IL-31, which result in pruritus, the inhibition of the gene expression of filaggrin, loricrin, involucrin, and lipid components of the skin barrier, or the activation of eosinophilia. The skin becomes dehydrated and is characterised by persistent pruritus, the integrity of the skin barrier is destabilised, and there is an increased likelihood of superinfection [63]. Research suggests that the balance of the immune response is a key factor in protecting the host against the development of atopic dermatitis, and consequently, regulation of the immune response is an effective method of alleviating AD in patients. At this point, it is worth noting that any intestinal dysbiosis results in altered immune responses. Therefore, the etiology of AD should also be sought when considering the disruption of the normal gut microbiota [34].

Studies have shown an association with gut dysbiosis in patients with atopic dermatitis. Xue et al. analysed the genetic link between the gut microbiome and atopic dermatitis. The gut microbiome data came from a large GWAS analysis of the MiBioGen consortium involving 18,340 individuals, including 24 cohorts for whole-genome genotypes and 16S faecal microbiome data. AD data were derived from well-defined AD data collected in the FinnGen biobank analysis, consisting of 218,476 individuals (5321 AD patients and 213,146 controls). The results showed that *Mollicutes*, *Clostridia*, *Bifidobacterium*, *Bifidobacteriales*, *Bifidobacteriaceae*, *Tenerticutes*, and *Christensenellaceae R 7 group* were negatively correlated with AD risk, while *Clostridiaceae_1*, *Bacteroides*, *Anaerotruncus*, *Bacteroidaceae*, *unknown genus, and Lachnospiraceae UCG001* had a positive correlation [60]. 

Wang et al. examined stool samples from 234 adults (104 AD patients and 130 controls). Their results indicated that, in the AD patient group, the microbiome was abundant in *Blautia*, *Butyricicoccus*, *Lachnoclostridium*, *Eubacterium_hallii_group*, *Erysi-pelatoclostridium*, *Megasphaera*, *Oscillibacter*, and *Flavonifractor*, while in the control group it was rich in *Romboutsia* and *Clostridi-um_sensu_stricto_1* [64].

Ye et al., analysing stool samples from 44 AD patients and 49 healthy controls aged 6–22 years, showed that the relative abundance of *Porphyromonadaceae*, *Blautia*, *Parabacteroides*, *Bacteroides ovatus*, *Bacteroides uniformis*, and *Prevotella stercorea* was significantly higher among AD patients than healthy controls, while the abundance of *Clostridium* and *P. stercorea* was relatively higher in AD patients than in healthy controls [65].

It is noteworthy that alpha diversity among patients with adult-onset atopic dermatitis (AOAD) is reduced, with *Escherichia—shigella* being the predominant group type. The relative level of *Bacteroides* pectinophilus was higher and the levels of *Agathobacter* and *Dorea* were relatively lower than in the healthy subjects and the chronic AD group. These results were obtained from analyses by Liu et al. through the analysis of stool samples from 10 healthy subjects, 12 AOAD patients, and 10 chronic AD patients [66].

Interestingly, the microflora of the group of AD patients accompanied by gastrointestinal symptoms differed from those of patients with simple AD. In patients with gastrointestinal symptoms expressed as epigastric stiffness or a feeling of fullness in the epigastrium, the microflora was richer in *Bacteroides*, while the proportion of *Prevotella* decreased compared to AD patients without gastrointestinal symptoms. This was shown in the study by Han et al. [67].

Researchers confirm that the appearance of the gut microbiota in early life is associated with age of onset, disease exacerbations, remission, severity, and atopic dermatitis phenotype [68]. A study of 1440 school-aged children by Hu et al. found that reduced alpha diversity of the gut microbiota was strongly associated with increased AD risk [69].

The influence of gut bacteria in the development of AD in children may begin even before birth, as dysbiosis in pregnant women is associated with poor immune system development in offspring. Sung et al., studying the gut microbiomes belonging to nine healthy infants and six infants with AD and their mothers, proved that the absence of *Akkermanisia miciniphila* in mothers and their children was associated with the absence of the appearance of AD [70].

In addition, Fan et al. studied 36 mother–offspring pairs. The results presented that mothers of infants and young children with AD had a higher abundance of *Candidatus_Stoquefichus* and *Pseudomonas* during pregnancy and that children with AD had a higher abundance of *Eubacterium_xylanophilum_group* at birth, *Ruminococcus_gauvreauii_group* after 1 year of age, and *UCG-002* after 2 years of age, and a lower abundance of *Gemella* and *Veillonella* after 2 years of age [71].

Moreover, as Melli et al. demonstrated in a study involving 81 children aged 5–11 years, the microflora of children with AD is characterised by a higher abundance of *C. difficile* and *Bifidobacterium* spp. In contrast, the numbers *of Eubacteria*, *B. fragilis*, *Lactobacillus* spp., *E. coli*, and *M. smithii* were lower in children with AD, irrespective of socio-economic status [72].

Kingkaw et al. evaluated one faecal sample each from 18 infants (11 healthy infants and 7 infants with AD). They used liquid chromatography and tandem mass spectroscopy for analysis. Their study showed that triosephosphate isomerase (TPI) in the *Bifidobacteriaceae* of the genus *Alloscardiovia* and demethylmenaquinone methyltransferase (DMM) in *Babcteroides* play metabolic functional roles associated with AD [73].

It is well known that the coexistence of AD with autism is not uncommon and that the gut microbiota plays a role in the development of both diseases. This knowledge prompted Hong et al. to determine the differences in the gut microflora of autistic patients with and without AD and the collation of gut microflora with organic acids in urine. Sixty-one autistic children (36 AD patients and 25 controls) were enrolled in the study. The alpha diversity of the gut microflora was lower in the AD group. AD patients showed a higher abundance of *Anaerostipes caccae*, *Eubacterium Hallii*, and *Bifidobacterium bifidum* compared to controls, while the control group had a higher abundance of *Akkermansia muciniphila*, *Roseburia intestinalis*, *Haemophilus parainfluenzae*, and *Rothia mucilaginosa* [74].

In Table 1, the authors collect selected studies on the gut microflora in patients with atopic dermatitis.

It is also worth focusing our attention on probiotics, as studies on their effects have shown that they can influence the incidence and development of atopic dermatitis and appear to be an effective therapeutic option for this dermatosis [75,76,77].

Kim et al. conducted a study in which they observed that the administration of a multispecies probiotic (*Bifidobacterium bifidum W23*, *Bifidobacterium animalis subsp. Lactis W52*, *and Lactococcus lactis W58*, Ecologic^®^ Panda) is associated with increased levels of short-chain fatty acids (SCFAs) and lactate and decreased levels of lactose and succinate, which may explain the protective effect of probiotics on the occurrence of AD [78].

Navarro et al. administered a probiotic combination of *Bifidobacterium longum*, *Bifidobacterium lactis*, and *Lactobacillus casei* to 50 paediatric patients aged 4 to 17 years with AD for 12 weeks. This led to an 83% reduction in SCORAD and a reduction in the use of topical steroids [79].

The positive effects of probiotics were also presented by Yoon et al. Their study involved 25 children who were given a probiotic mixture that included *Lactobacilli* and *Bifidobacteria* strains for 4 weeks. The SCORAD index decreased significantly; alpha diversity did not change significantly, while beta diversity increased [80].

Choy et al. conducted a clinical evaluation and analysis of stool samples from 24 children with AD before and after taking the new symbiotic formulation for eight weeks. After eight weeks of therapy, there was a significant improvement in the Eczema Area and Severity Index (EASI) and no adverse effects were observed. The relative abundance of key microbial agents including *Lactobacillus acidophilus* and *Bacteroides fragilis* increased significantly [81].

Wang et al. report that after a sustained 8-week intake of a novel E3 probiotic formulation (containing a prebiotic, a probiotic, and a postbiotic), AD patients showed an increased relative abundance of *Clostridium*, *Fecalibacterium*, *Lactobacillus*, *Romboutsia*, and *Streptococcus*; a lower relative abundance of *Collinsella*, *Bifidobacterium*, *Fusicatenibacter*, and *Escherichia-Shigella*; and a composition and structure of the gut microbiome resembling healthy subjects. Patients with mild AD were more likely to respond to probiotic treatment, while species richness was significantly increased among responders, regardless of disease severity. Forty-one AD patients participated in the analysis [82].

Faecal microbiota transplantation (FMT) may be an effective method for restoring intestinal homeostasis. Mashiah et al. performed the first evaluation of the efficacy and safety of FMT in humans, specifically on nine adults with moderate-to-severe AD. Four sessions of FMT were performed. The response rate was 77%. FMT resulted in significant clinical improvement compared to baseline. No adverse effects were reported [83].

Recently, a clinical case report was published in which a 15-year-old boy with AD underwent microflora smuggling transplantation (WMT). WMT is a variation of FMT that involves taking a stool sample from a healthy donor, centrifuging it repeatedly, sedimenting the microbial precipitate, making a suspension with saline, and administering it in this form to the patient’s lower gastrointestinal tract. After three months of treatment, which consisted of three WMT sessions, the patient’s pruritus was controlled and there was a marked improvement in skin lesions, with SCORAD, EASI, NRS, and DLQI scores decreasing markedly from baseline [84].

However, further studies are needed to confirm the efficacy of FMT and related methods in the treatment of AD.

### 3.2. Psoriasis

Psoriasis is a chronic inflammatory skin disease. Its symptoms include the appearance of sharply demarcated red plaques. These lesions are most commonly found on the scalp, trunk, and upright surfaces of the extremities [32,85]. The most recent World Health Organisation report states that the prevalence of psoriasis is increasing, ranging from 1.5% to 5% in developed countries [86]. In the course of psoriasis, abnormalities in the functioning of other systems in addition to skin disorders have also been reported, suggesting that this dermatosis is not just a skin disease [87]. Psoriasis is thought to be caused by environmental interactions and immune dysregulation in genetically susceptible individuals, and its course is characterised by relapses and remissions [88]. 

Psoriasis is caused by chronic inflammation that leads to the uncontrolled growth of keratinocytes and their abnormal differentiation. When analysing psoriatic plaques histologically, it is observed that epidermal proliferation coexists with infiltrates composed of dermal dendritic cells, neutrophils, macrophages, and T lymphocytes [89]. Inflammation is also mediated by molecules such as TNF-α, IL-17, and Il-6 [90]. A number of studies show that the pathogenesis of psoriasis is also influenced by a disruption of the gut–skin axis, in which the gut microbiome, and more specifically its dysbiosis, plays a major role.

Wang et al. demonstrated that there is significant dysbiosis in the microbiome of psoriasis patients. Stool samples from 28 psoriasis patients and 21 healthy individuals were used for the study. The microbiome of psoriasis patients was characterised by a higher abundance of *Bacteroidetes* with a lower abundance of *Proteobacteria* compared to the control group. At the genus level, among psoriasis patients, *Lactobacillus* and *Dialister* were relatively more abundant, while *unidentified_Enterobacteriaceae*, *unidentified_Lachnospiraceae*, *Romboutsia*, *Subdoligranulum*, *unidentified_Erysipelotrichaceae*, and *Dorea* were relatively less abundant compared to the control group [91].

Hidalgo-Cantabrana et al. examined, via 16S rRNA sequencing, stool samples from 39 adults (19 psoriasis patients and 20 healthy individuals). They concluded that the gut microflora of psoriasis patients was characterised by lower diversity. The study also found that, as expected, the core microflora of both study groups included the following bacterial types: *Actinobacteria*, *Bacteroidetes*, *Firmicutes, and Proteobacteria*. However, their abundance differed between the psoriasis patients and healthy groups. The number of *Bacteroidetes* and *Proteobacteria* was markedly reduced in the psoriasis patient group compared to the control group, while *Actinobacteria* and *Firmicutes* were significantly increased. Furthermore, among the *Ruminococcaceae* family, which was significantly higher among psoriasis patients, *Ruminococcus* and *Subdoligranulum* genera were relatively elevated, while *Faecalibacterium* was lower [92].

Tan et al. investigated the intestinal microbiota by analysing faecal samples from 28 individuals (14 patients struggling with psoriasis and 14 healthy patients) and concluded that there was a decrease in the abundance of *Akkermansia muciniphila* species among psoriasis patients, compared to controls [93]. This species is responsible for intensifying intestinal integrity [94]. This study also showed that the abundance of *Clostridium citroniae* species was elevated in patients with psoriasis [93].

Another study, conducted by Schade et al., showed an increase in the abundance of the genus *Dialister* and species *of Prevotella*, a decrease in the abundance of the genera *Ruminococcus*, *Blautia* and *Lachnospira*, and a decrease in the abundance of the species *Akkermansia muciniphila* among psoriasis patients, compared to the control group. The study included 45 participants (21 patients with psoriasis and 24 constituting the control group) [95].

Zhang et al. analysed stool samples from 30 people with psoriasis and 30 healthy controls. Their study yielded the following result: the relative abundance of *Faecalibacterium* and *Megamonas* was higher among those struggling with psoriasis. Furthermore, the researchers observed that the IL-2 receptor, which is a marker of T-lymphocyte activation and is significantly elevated in psoriasis, showed a positive correlation *with Phascolarctobacterium*, but a negative correlation with *Dialister*. It is emphasised that the increased abundance of *Phascolarctobacterium* may be considered as a factor involved in the inflammatory response and pathogenesis of psoriasis [90].

Yu et al., using Mendelian randomisation, analysed data on 4510 patients with psoriasis and 212,242 control subjects. Based on the analyses, they found that *Lactococcus*, *Ruminiclostridium 5*, and *Eubacterium fissicatena* present in the intestinal microflora were risk factors for psoriasis, while *Odoribacter* showed a protective effect against psoriasis [96].

Wen et al. studied the faecal microflora of 32 untreated patients with plaque psoriasis, 17 healthy spouses, and 15 healthy controls. They found that the gut flora of psoriasis patients was significantly enriched in *Escherichia coli* compared to healthy subjects and healthy spouses. Furthermore, among psoriasis patients, *Firmicutes* decreased and *Bacteroidetes* increased, resulting in a decreased F/B ratio. The microbiota in patients with severe psoriasis differed from that of patients with milder psoriasis [97].

Zang et al. conducted a two-sample Mendelian randomisation study to assess the possible association between gut microflora and psoriasis. The study showed that *Bacteroidetes* and *Prevotella9* were nominally associated with a lower risk of psoriasis, while *Eubacterium Fissicatena group 9* was associated with a higher risk of psoriasis [98].

Xiao et al. performed a comprehensive identification of the characteristic gut microbiota composition, genetic function, and metabolites of psoriasis patients. The researchers analysed DNA from stool samples from 45 individuals (30 psoriasis patients and 15 controls). The intestinal microflora in psoriasis patients was characterised by an increased abundance of *Actinobacteria*, *Verrucomicrobia* and *Firmicutes* types, as well as *Rosebusia*, *Megamonas*, *Bifidobacterium*, *Bacteroides*, and *Faecalibacterium* genera. In addition, a reduced abundance of the *Proteobacteria*, *Euryarchaeota*, and *Bacteroides* genera, as well as the *Prevotella*, *Eubacterium*, and *Alistipes* genera, was noted among those with this dermatosis. Another observation that followed this study was that levels of hydrogen sulphide, haemicellulose, hyaluronate, isobutyrate, and isovalerian were markedly deregulated among psoriasis patients [99].

Sun et al., in their study that included psoriasis patients aged 18–60 years and a control group, showed that in 85.5% of psoriasis patients, at least one gastrointestinal symptom occurred. This compares with 58.1 per cent in the control group. Furthermore, it was also noted that the abundance of the family *Ruminococcaceae*, genus *Coprococcus_1*, and *Blautia* decreased with strain in favour of psoriasis [88].

It should be noted that a reduced *Firmicutes/Bacteroidetes* ratio is also observed in the course of psoriasis, which is associated with some comorbidities with psoriasis such as metabolic syndrome and shows a positive correlation with the PASI score [100,101].

Table 2 highlights the selected studies on the intestinal microflora in patients with psoriasis.

Although bespoke regulation of the gut microflora is not part of psoriasis therapies, increasing evidence suggests that it may have a potential role in alleviating the symptoms of this dermatosis. Probiotics are known to have an impact on overall skin condition.

Gueche et al. showed that administration of the probiotic *Lactobacillus paracasei* NCC2461 to humans over a two-month period led to a decrease in transepidermal water loss and skin sensitivity to high levels of transforming growth factor beta (TGF-β) [102]. In addition, the gut microbiome also influences skin allostasis through both innate and acquired immunity [103].

There are an increasing number of studies indicating the efficacy of probiotics in the treatment of psoriasis. A probiotic mixture was tested in the treatment of psoriasis on 90 adult plaque psoriasis patients randomly assigned to probiotic and placebo groups. The intervention proved to be effective up to 6 months afterwards, with fewer relapses in the group treated with the probiotic mixture [104].

Furthermore, supplementation for 6–8 weeks with *Bifidobacterium infantis* strain 35,624 was associated with a decrease in CRP and TNF-α among psoriasis patients [105].

As reported in a study by Moloudi et al. the use of *Lactobacillus* strains for 8 weeks was associated with positive effects on oxidative stress parameters and inflammation (decreases in hs-CRP and MDA levels and an increase in TAC). Moreover, probiotics also influenced the reduction in disease symptoms (reduction in PASI and PSS) [106].

Lin et al., on a group of 26 patients with psoriasis, tested the effect of *Bacteroides fragilis* BF839. The probiotic was supplemented for 12 weeks with concomitant anti-psoriasis treatment. The study showed a reduction in PASI. An adverse effect in the form of constipation occurred in one patient [107].

A recently published study by Buhaș et al. showed that patients taking spore-based probiotics and prebiotics for 12 weeks achieved better results in the measurement of psoriasis area and severity index, dermatological quality of life index, inflammatory markers, and skin thickness compared to patients not receiving supplementation. In addition, the gut microflora changed favourably towards an anti-inflammatory profile. The probiotics used in the study were *Bacillus indicus*, *Bacillus subtillia*, *Bacillus coangulans*, *Bacillus licheniformjis*, and *Bacillus clausii*, while the prebiotics given to patients were fructooligosaccharides, xyloligosaccharides, and galactooligosaccharides [108].

Choy et al. analysed the use of a novel E3 probiotic formulation (prebiotic + probiotic + postbiotic) in patients with psoriasis. After 8 weeks of therapy, the dermatological quality of life index and the psoriasis fiducial and severity index improved significantly [109].

Further research is needed to clarify the benefits of probiotics and prebiotics in the treatment of this dermatosis and to determine the effective dose and combination to implement them as a routinely recommended therapy for patients struggling with this dermatosis.

### 3.3. Acne

Acne is a chronic and widespread inflammatory skin disease involving hair and sebaceous units that affects, predominantly, relatively young people and has a serious impact on patients’ quality of life, causing low self-esteem, difficulties in social interaction, and psychological distress [110]. The disease manifests as inflammatory lesions usually located on the face, arms and chest, and non-inflammatory lesions such as open or closed comedones [111,112]. Severe forms of the disease can lead to disfigurement and scarring [5]. Acne vulgaris is the most common skin disease in the Western world and can affect between 79% and 95% of adolescents [113].

The pathogenesis of acne vulgaris is multifactorial and complex. It includes an increased production of skin sebum, androgen stimulation of the sebaceous glands and their subsequent proliferation, obstruction of the ducts of excretion due to increased exfoliation of keratinocytes, proliferation, abscesses on the skin of *Cutibacterium acnes* (formerly described as *Propionibacterium acnes*) and the resulting inflammatory response [114]. The human gut, and more specifically the microorganisms that inhabit it, also play a significant role in the pathogenesis of acne, primarily through modification of the mTOR pathway and through increased permeability of the intestinal barrier [115,116,117]. Although the gastrointestinal microbiome is only one of many factors contributing to acne, in the case of acne vulgaris, it has an undeniable impact on skin conditions [53].

A study performed by Deng et al. on 43 acne patients and 43 controls showed that acne patients have a distinct composition of the gut microbiome compared to controls. At the cluster level, the abundance of *Firmicutes* among the patients was lower, while the abundance of *Bacteroides* was higher, which is an enterotype of the Western diet. In addition, the gut microflora of acne patients was impoverished in genera such as *Clostridia*, *Clostridiales*, *Lachnospiraceae*, and *Ruminococcaceae*, which are characterised by beneficial effects due to their ability to produce SCFAs [118].

Yan et al. analysed faecal samples from 31 patients with acne vulgaris and 31 patients who were controls. Their results showed, at the cluster level, a decrease in *Actinobacteria* and an increase in *Proteobacteria*, while at the genus level there was a decrease in *Bifidobacterium*, *Butyricicoccus*, *Lactobacillus*, *Coprobacillus*, and *Allobaculum* in patients in the study group compared to the control group [119]. It is known that Lactobacillus and Bifidobacterium are species that balance the intestinal microflora by fermenting unabsorbed oligosaccharides in the upper intestine, enhance the tightness of the intestinal barrier, and suppress the response of T helper lymphocytes, B lymphocytes, and cytokine production [120,121].

Cao et al. assessed the causal relationship between gut microflora and acne using Mendelian randomisation. Their analyses showed that the *Ruminococcus torques* group was protective against acne. Furthermore, four other types of gut microflora, including those from the *Candidatus soleaferrea* group and *Eubacterium coprostanoligenes* showed suggestive protective effects against acne. In contrast, *Allisonella* and *Bacteroides* were responsible for exacerbating acne [122].

Interestingly, Deng et. al. also showed that there are gender differences in the gut microbiota during the course of acne vulgaris. Stool samples from male patients were characterised by a lower abundance of 18 bacterial genera (*Butyricicoccus*, *Clostridium sensu stricto*, *Ruminococcus*, *Blautia*, *Clostridiales*, *Bacillus*, *Faecalibaculum*, *Lachnospiracea incertae sedis*, *Lysinibacillus*, *Peanibacillus*, *Aerococcus*, *Alkaliphilus*, *Carnobacterium*, *Lactococcus*, *Oceanobacillus*, *Gemmiger*, *Exiguob Acterium*, *Pseudomonas*, *Enterococcus*, and *Bilophila*), compared to the control group, while women struggling with acne showed a decrease in *Oscillibacter* and *Odoribacterin* and an increase in *Clostridium sensu stricto*. It is also noteworthy that abnormal amino acid metabolism was observed in women with established acne, while abnormal fatty acid metabolism was observed in men [123].

Sivamiani et al. investigated the association of the gut microbiome with acne and, more specifically, with inflammatory and non-inflammatory lesions occurring in the course of this dermatosis in 17 participants. A positive correlation with the occurrence of non-inflammatory lesions (open or closed comedones) was demonstrated by *Actinomyces naeslundii str Howell 279*, *Bifidobacterium dentium*, *Intestinibacter bartlettii DSM 16795*, and *Eubacterium sp AM28-29*. In contrast, the following strains had a negative correlation with the appearance of non-inflammatory lesions: *Blautia obeum ATCC29174*, *Massilioclostridium coli*, *Schaalia odontolytica*, *Adlercreutzia equolifaciens subsp celatus*, and *Butyricicoccus* sp *GAM44*. Strains positively correlated with inflammatory lesions were *Coprococcus* sp *AF16-22*, *Butyrivibrio crossotus DSM 2876*, *Clostridium sp AF23-8*, *Escherichia coli KTE51*, *Akkermansia muciniphila ATCC BAA-835*, *Bilophila wadsworthia 316*, and *Methanobrevibacter smithii DSM2375*. A negative correlation with inflammatory changes was confirmed for *Coprococcus sp ART55-1* and *Alistipes senegalensis JC50* [124].

In Table 3, the authors collect the selected studies on the gut microflora in patients with acne. It is worth emphasising that further studies should be carried out to identify the intestinal flora of acne patients more precisely.

It is worth noting that Thompson et al. conducted a study involving eight participants with moderate-to-severe acne and eight participants in a control group. At the start of the study, stool was collected from all participants and the acne treatment group was then treated with minocycline for 4 weeks. The intestinal microflora in the acne patients before antibiotic therapy compared to the control group without acne was deficient in *Lactobacillus iners*, *Lactobacillus zeae*, and *Bifidobacterium Animalis*. After antibiotic therapy, patients with acne had a decrease in *Lactobacillus salivarius*, *Bifidobacterium adolescentis*, *Bifidobacterium pseudolongum*, *Bifidobacterium breve*, and *Akkermansia mucinophila* compared to the healthy control group. Furthermore, patients had an increase in faecal *Bacteroidetes* after minocycline therapy, which implied a decrease in the *Firmicutes* to *Bacteroidetes* (F/B) ratio. This small study highlighted the presumed importance of probiotic use [125].

To date, there are few studies on the effect of probiotics on acne-prone skin [126]. 

A study by Kim et al. involving 36 patients showed that the consumption of a fermented dairy drink containing *Lactobacillus* bacteria for 12 weeks improved the clinical symptoms of acne, leading to a reduction in the total number of lesions by significantly reducing sebum secretion [127].

Very significant and interesting results came from a recent study by Irshad et al. The study involved 75 patients with acne. Patients were divided into three groups: group A received azithromycin, group B probiotics, and group C both azithromycin and probiotics. Therapy with the above-mentioned treatments lasted 3 months. After this time, all patients showed a significant improvement in the number of lesions. In group A, the average number of lesions decreased by 83.3%, in group B by 84.4%, and in group C by 90.3%. This shows that probiotics have the same efficacy as azithromycin, and that therapy administered simultaneously with azithromycin and a probiotic gave the best relative treatment effect [128].

Jung et al. randomly assigned 45 female acne sufferers to one of three groups: using a probiotic, using a minocycline, and using both a minocycline and a probiotic. After completion of the analyses, conclusions were reached that probiotics could be considered as a potential therapeutic option or adjunct in the treatment of acne vulgaris. Furthermore, probiotics minimised the appearance of side effects resulting from antibiotic therapy [129].

There is a great need for further research into modifying the gut microbiota with probiotics to reduce acne lesions.

### 3.4. Alopecia Areata

Alopecia areata (AA) is an autoimmune disease characterised by the partial or complete sudden loss of hair from the scalp or other hairy parts of the body without scarring [130]. The fact that scarring is not observed in the course of this dermatosis is related to the fact that the hair follicle is not destroyed, but preserved [131]. The incidence is, on average, 2% worldwide in the general population, with no difference between age, gender or ethnicity [132]. The first manifestation of this dermatosis usually occurs before the age of 30 [63]. AA can imply psychological suffering for the patient and a reduced quality of life, especially when areas of the body such as the scalp, chin, moustache, eyelashes, or eyebrows are affected [133]. It is widely accepted that the interplay of genetic and environmental factors is important in the onset and progression of this disease [134]. Increasingly, there is a view that, assuming host genetic susceptibility, AA occurs through oxidative stress, neuropsychological factors, disruption of the inflammatory pathway, and pathogens, in combination with co-morbidities and micro-ecological imbalances [135,136].

There is clinical and experimental evidence indicating that AA is a manifestation of an autoimmune attack on the hair follicles, which causes inflammation of the hair follicles [137,138,139]. Maslovsky and Macay, in their study, announced that the gut microflora contributes to the higher incidence of autoimmune diseases in developed countries [140]. To date, there have been few studies that demonstrate a link between the gut microflora and the pathogenesis of alopecia, but those that have been published are worth reviewing.

Moreno-Arrones et al. analysed whether and what differences in gut bacterial composition exist in alopecia areata patients compared to healthy individuals. The study included 15 patients struggling with AA and 15 control subjects. There were no statistical differences in either alpha diversity or beta diversity between the patients and the control group. Patients with alopecia showed an increased presence of *Holdemania filiformis*, *Erysipelotrichacea*, *Lachnospiraceae*, *Parabacteroides johnsonii*, *Clostridiales vadin BB60 group*, *Bacteroides Eggerthii*, and *Parabacteroides distasonis*. In addition, a predictive model based on the number of *Parabacteroides distasonis* and *Clostridiales vadin BB60* group bacteria correctly predicted the condition in 80% of patients [141].

The study performed by Brzychcy et al. involved 25 adult patients suffering from AA. The aim of the study was to describe, for the first time, the characteristics of the gut microbiome of AA patients on the basis of stool samples. These patients were shown to have four main genera forming the core of the microbiome—*Lachnoclostridium*, *Eubacterium*, *Streptococcus* and *Bifidobacterium*—and three major types: *Firmiutes*, *Proteobacteria* and *Actinobacteria*. The composition of the bacterial biodiversity suggested a loss of overall richness and a reduction in taxonomic diversity in all samples [13].

Lu et al. examined stool samples from 33 AA patients and 35 controls. They found no statistically significant differences in alpha diversity. The overall gut microbial communities in the AA patients differed from the control group. Three OUT biomarkers associated with AA were identified: OUT1237 (*Achromobacter*), OTU257 (*Megasphaera*), and OUT1784 (*Lachnospiraceae Incertae Sedis*) [142].

Rangu et al. conducted a cross-sectional study of the gut microbiome of paediatric patients with AA based on the analysis of stool samples from 41 children with AA. Healthy siblings of these patients were also included in the study. A comparison of alpha and beta diversity yielded a small but statistically significant difference between those with AA and those in the control group. Furthermore, the relative abundance of one species, *Ruminococcus bicirculans*, was reduced in patients with alopecia areata compared to controls. The analysis of gene orthologue abundance identified 20 orthologues that differed between groups, including spore germination genes and genes responsible for metal transport [143].

In Table 4, the authors collect the studies on the intestinal microflora in patients with alopecia areata.

To date, there are no studies on the use of probiotics in the treatment of alopecia areata. However, one publication by Rebello D. et al. is noteworthy, in which two patients were described following faecal microflora transplantation (FMT). The primary goal for performing FMT in these patients was to cure them of recurrent *C. difficile* infection (CDI). Both patients also suffered from alopecia areata as a co-morbid condition, and tried to combat it with various dedicated therapies without success. After FMT, the patients experienced hair regrowth. This is an extremely important publication, offering hope for an effective AA therapy for people in whom other treatments have failed. However, further research is needed to introduce alternative treatment options, such as the aforementioned FMT, into the treatment of patients struggling with AA [144].

## 4. Conclusions

The increasing prevalence of dermatological diseases poses a challenge both to public health and to patients themselves. Dermatoses are often a cause of reduced quality of life, satisfaction with appearance, and sense of self-confidence for the patient. This can imply impaired social functioning and lowered mood, which is why it is so important to introduce effective therapies to address the symptoms of skin conditions. Researchers are constantly searching for further factors and pathomechanisms leading to dermatological diseases and, consequently, new therapeutic targets. In recent years, there has been a growing interest in the influence of the gut microbiome and, more specifically, its dysbiosis, on the pathogenesis of diseases, including skin disorders. After analysing the available data, the authors conclude that there is a growing body of scientific evidence supporting a causal link between the gut microbiota and dermatological conditions such as atopic dermatitis, psoriasis, acne, and alopecia areata. However, there is a great need for further research, especially focusing on functional features of the microbiome, such as transcriptomics, lipidomics, and the measurement of secondary metabolites. This will allow the design of effective therapies that, through the modulation of the intestinal microflora, will lead to the effect so desirable for many patients and often unattainable with previously available methods—the resolution of skin lesions. To date, relatively few studies have been carried out to evaluate the effect of probiotics on the treatment of AD, psoriasis, acne, or AA, but those that do exist are optimistic. Furthermore, the use of FMT also appears to have a beneficial effect in remodelling the intestinal microflora, leading to an alleviation of dermatoses. Also noteworthy is the fact that, unlike many of the currently available methods used by dermatologists in their practice, interventions that modulate the gut microbiome are extremely safe and have a low risk of side effects. The authors believe that therapies targeting the remodelling of the gut microbiota represent the future of the treatment of AD, psoriasis, acne, and AA. However, further research is needed to clarify the benefits of probiotics and prebiotics in the treatment of dermatoses and to determine effective doses and combinations to implement them as a routinely recommended therapy for patients struggling with skin diseases.

## Figures and Tables

**Table 1:** Selected studies on the intestinal microflora in patients with atopic dermatitis.

Research	Year	Methodology	Key Results	References
Kingkaw A. et al.	2020	Analysis of stool samples from 18 infants (11 infants without AD and 7 infants with AD). Analysis was performed using liquid chromatography–tandem mass spectrometry.	Triosephosphate isomerase (TPI) in the *Bifidobacteriaceae* of the genus *Alloscardiovia* and demethylmenaquinone methyltransferase (DMM) in *Babcteroides* play metabolic functional roles related to the occurrence of AD.	[73]
Melli L. et al.	2020	Stool samples from 81 children aged 5–11 years (23 AD patients and 58 controls) were analysed using real-time PCR.	Children with AD had higher abundance of *C. difficile* and *Bifidobacterium* spp. Children with AD showed lower abundance of *Eubacteria*, *B. fragilis*, *Lactobacillus* spp., *E. coli*, and *M. smithii*.	[72]
Ye S. et al.	2020	Analysis of stool samples from 93 individuals (44 AD patients and 49 controls) aged 6–22 years. 16S rRNA sequencing.	AD patients had lower alpha diversity than healthy control patients. The relative abundance of *Blautia*, *Bacteroides ovatus*, *Porphyromonadaceae*, *Bacteroides uniformis*, and *Parabacteroides* was significantly higher among AD patients than controls. *Prevotella stercorea* and *Clostridium* counts were higher in healthy controls compared to patients with AD. *Bacteroidaceae* and *Porphyromonadaceae* may act as possible biomarkers related to AD diagnosis.	[65]
Hu C. et al.	2021	Prospective cross-sectional study analysing stool samples from 1440 children aged 10 years by 16S rRNA sequencing.	Alpha diversity of faecal microflora was associated with reduction in eczema risk. The *species Lachnospiraceae*, *Ruminococcaceae_UCG—005*, and *Christensenellaceae_R-7_group* were associated with a reduced risk of eczema.	[69]
Fan X. et al.	2022	Faecal samples of 36 mother–offspring pairs were analysed for 2 years postpartum. Samples were subjected to sequencing using a platform.	Mothers of infants and young children with AD had increased abundance of *Candidatus_Stoquefichus* and *Pseudomonas* during pregnancy. In infants and young children with AD, a higher abundance of *Eubacterium_xylanophilum_group* at birth, *Ruminococcus_gauvreauii_group* after 1 year, and *UCG-002* after 2 years, and a lower abundance of *Gemella* and *Veillonella* after 2 years were observed.	[71]
Han C. et al.	2022	Stool samples from 27 patients (20 AD patients and 7 controls) were analysed using 16S rRNA sequencing. AD patients were divided into groups with the presence of symptoms of epigastric fullness, epigastric stiffness, and no gastrointestinal symptoms.	AD patients with gastrointestinal symptoms have a gut microbiome abundant in *Bacteroides*, but poorer in *Prevotella* compared to AD patients without gastrointestinal symptoms.	[67]
Hong R. et al.	2022	Stool samples from 61 autistic children (36 with AD and 25 without AD) were analysed. The gut microflora was sequenced using shotgun metagenomic sequencing.	The diversity of alpha gut microflora was lower in the AD group. People with AD showed higher abundance of *Anaerostipes caccae*, *Eubacterium Hallii*, and *Bifidobacterium bifidum* compared to the control group, while the control group had higher abundance of *Akkermansia muciniphila*, *Roseburia intestinalis*, *Haemophilus parainfluenzae*, and *Rothia mucilaginosa*.	[74]
Liu T. et al.	2022	Analysis of stool samples of 10 healthy patients, 12 patients with adult-onset AD (AOAD), and 10 persistent AD patients. 16S rRNA sequencing.	Alpha diversity of gut microflora in AOAD patients was reduced. The most dominant type of AOAD was *Escherichia-shigella* (15.8%). Compared to healthy volunteers and patients with chronic AD disease, the relative levels of the *Bacteroides pectinophilus* group in AOAD were significantly increased while *Agathobacter* and *Dorea* in AOAD patients were significantly decreased.	[66]
Sung M. et al.	2022	Analysis of stool samples 30 days and 12 months postpartum from 15 mother–child pairs (6 infants with AD and 9 healthy infants). 16S rRNA sequencing.	*Akkermansia muciniphila* was detected in healthy infants and their mothers. Occurrence of 12 species that differed in AD infants compared to healthy infants. Six species were significantly different in mothers of AD infants compared to mothers of healthy infants.	[70]
Wang Y. et al.	2023	Stool samples from 234 adults (104 AD patients and 130 controls) were examined by 16S rRNA sequencing.	The microbiome of the control group was abundant in *Romboutsia* i *Clostridi-um_sensu_stricto_1*, whereas the microbiome of AD patients was rich in *Blautia*, *Butyricicoccus*, *Lachnoclostridium*, *Eubacterium_hallii_group*, *Erysi-pelatoclostridium*, *Megasphaera*, *Oscillibacter*, and *Flavonifractor*.	[64]
Xue Y. et al.	2023	The gut microbiome data came from a large GWAS analysis of the MiBioGen consortium comprising 18,340 individuals, including 24 cohorts for whole-genome genotypes and 16S faecal microbiome data; AD data came from well-defined AD data collected in a FinnGen biobank study consisting of 5321 AD patients and 213,146 controls. The inverse variance-weighted method, weighted median, MR-Egger, Cochran’s Q test, and MR Steiger’s test were used.	*Tenericutes*, *Mollicutes,* *Clostridia*, *Bifidobacteriaceae*, *Bifidobacteriales*, *Bifidobacterium*, *and Christensenellaceae R 7* group were negatively correlated with the risk of AD; *Clostridiaceae 1*, *Bacteroidaceae*, *Bacteroides*, *Anaerotruncus*, the unknown genus, and *Lachnospiraceae UCG001* were positively correlated with the risk of AD; MR Steiger’s test showed a potential causal relationship between the above intestinal flora and AD.	[60]

**Abbreviations:** AD = atopic dermatitis; PCR = polymerase chain reaction; rRNA = ribosomal RNA; AOAD = adult-onset atopic dermatitis; MR = Mendelian randomization.

**Table 2:** Selected studies on the intestinal microflora in patients with psoriasis.

Research	Year	Methodology	Key Results	References
Tan L. et al.	2017	Stool samples from 28 individuals (14 psoriasis patients and 14 controls) were analysed by 16S rDNA sequencing.	At the species level, *Akkermnasia muciniphila* abundance was reduced, while *Clostridium citroniae* abundance was increased in psoriasis patients’ gut microflora compared to controls.	[93]
Hidalgo-Cantabrana C. et al.	2019	Analysis of stool samples from 39 individuals (19 psoriasis patients and 20 controls) using the 16S rRNA gene sequencing method.	The gut microflora of patients with psoriasis was characterised by lower diversity compared to the control group. The number of *Actinobacteria* and *Firmicutes* was increased in patients with psoriasis compared to the control group. The number of *Bacteroidetes* and *Proteobacteria* was reduced in patients with psoriasis compared to controls. Among the family *Ruminococcaceae*, which was significantly higher among patients with psoriasis, the genera *Ruminococcus* and *Subdoligranulum* were relatively elevated, while *Faecalibacterium* was lower.	[92]
Sun C. et al.	2021	An epidemiological study was conducted on the differences in gastrointestinal discomfort symptoms between psoriasis patients and controls. 16S rRNA sequencing of faecal samples from psoriasis patients treated and untreated with NB-UVB was performed.	At least one gastrointestinal symptom occurred in 85.5% of psoriasis patients compared to 58.1% of controls. Transient flatulence and constipation correlated with the presence of psoriasis. The abundance of the family *Ruminococcaceae,* genus *Coprococcus_1*, and genus *Blautia* decreased with improvement in skin symptoms.	[88]
Xiao S. et al.	2021	DNA from stool samples from 45 individuals (30 patients with psoriasis and 15 representing the control group) was analysed.	The microflora of patients with psoriasis was characterised by a higher abundance of the types *Firmicutes*, *Actinobacteria* and *Verrucomicrobia* and the genera *Faecalibacterium*, *Bacteroides*, *Bifidobacterium*, *Megamonas*, and *Roseburia*, and a reduced abundance of the types *Bacteroidetes*, *Euryarchaeota*, and *Proteobacteria* and the genera *Prevotella*, *Alistipes*, and *Eubacterium*. The levels of five metabolites (haemicellulose, hyaluronate, isobutyrate, isovalerian, and hydrogen sulphide) were deregulated in the psoriasis group.	[99]
Zhang X. et al.	2021	Examination of stool samples from 60 individuals (30 patients with psoriasis and 30 controls) via 16S rRNA sequencing.	Abundance of *Faecalibacterium* and *Megamonas* increased in patients with psoriasis. IL-2 receptor showed a positive association with *Phascolarctobacterium* and a negative association with the *Dialister* group.	[90]
Schade L. et al.	2022	Examination of stool samples from 45 participants (21 participants with psoriasis and 24 healthy controls). 16S rRNA sequencing.	Increase in the abundance of the genus *Dialister* and species *Prevotella* among psoriasis patients compared to controls. Decrease in the abundance of the genera *Ruminococcus*, *Lachnospira*, and *Blautia* and a decrease in the abundance of the species *Akkermansia muciniphila* among psoriasis patients compared to controls.	[95]
Wang X.	2022	Stool samples from 28 psoriasis patients and 21 healthy controls were analysed by 16S rRNA sequencing.	The microbiome of psoriasis patients was characterised by a higher abundance of *Bacteroidetes* and lower abundance of *Proteobacteria* compared to the control group. At the genus level, among psoriasis patients, *Lactobacillus* and *Dialister* were relatively more abundant, while *unidentified_Enterobacteriaceae*, *unidentified_Lachnospiraceae*, *Romboutsia*, *Subdoligranulum*, *unidentified_Erysipelotrichaceae*, and *Dorea* were relatively less abundant compared to the control group.	[91]
Wen C. et al.	2023	The faecal microflora of 32 psoriasis patients, 17 healthy spouses, and 15 healthy controls was analysed. The method used was metagenomic gene sequencing.	The intestinal microflora of psoriasis patients was abundant in *Eschericia coli* compared to healthy subjects and healthy spouses. In the intestinal flora of psoriasis patients, it was noted that *Firmicutes* decreased and *Bacteroidetes* increased.	[97]
Yu N. et al.	2023	Data from the MiBioGen study and the FinnGen database resource, which included 4510 psoriasis cases and 212,242 control subjects. Data were processed using Mendelian randomization.	The presence of *Lactococcus*, *Ruminiclostridium 5, and Eubacterium fissicatena* in the gut microbiota was found to be a risk factor for psoriasis, while *Odoribacter* showed a protective effect against psoriasis.	[96]
Zang C. et al.	2023	MR was performed in two trials to assess the multiscale GWAS summary data sets.	*Bacteroidetes* and *Prevotella9* play a protective role in psoriasis risk. The *E. fissicatena* group is a possible risk factor for psoriasis.	[98]

**Abbreviations:** GWAS = genome-wide association study; NB-UVB = narrow-band ultraviolet B; MR = Mendelian randomization.

**Table 3:** Selected studies on the intestinal microflora in patients with acne.

Research	Year	Methodology	Key Results	References
Deng Y. et al.	2018	Analysis of the gut microflora of 43 acne patients and 43 control patients by sequencing the hypervariable V3-V4 regions of the 16S rRNA gene.	There are clear differences between acne patients and control subjects. At the cluster level, *Firmicutes* abundance was lower and *Bacteroidiain* abundance was higher among people with acne. The microflora of acne subjects was characterised by relatively low abundance of the genera *Clostridia*, *Clostridiales*, *Lachnospiraceae*, and *Ruminococcaceae.*	[118]
Yan H.-M. et al.	2018	Stool samples were analysed by 16S rRNA sequencing. Samples came from 31 patients with acne vulgaris and 31 controls.	At the phylum level, there was a decrease in the abundance of *Actinobacteria* and an increase in the abundance of *Proteobacteria* in patients with acne compared to controls. At the genus level, there was a decrease in the abundance of *Bifidobacterium*, *Butyricicoccus*, *Coprobacillus*, *Lactobacillus*, and *Allobaculum*.	[119]
Huang Y. et al.	2021	Analysis of the gut microflora of 43 acne patients and 43 control patients by sequencing the hypervariable V3-V4 regions of the 16S rRNA gene.	There are gender differences in the gut microbiota during the course of acne vulgaris. Faecal samples of male patients were characterised by a lower abundance of 18 bacterial genera (*Butyricicoccus*, *Clostridium sensu stricto*, *Ruminococcus*, *Blautia*, *Clostridiales*, *Bacillus*, *Faecalibaculum*, *Lachnospiracea incertae sedis*, *Lysinibacillus*, *Peanibacillus*, *Aerococcus*, *Alkaliphilus*, *Carnobacterium*, *Lactococcus*, *Oceanobacillus*, *Gemmiger*, *Exiguob Acterium*, *Pseudomonas*, *Enterococcus*, *Bilophila*), compared with the control group. Women struggling with acne showed a decrease in *Oscillibacter* and *Odoribacterin* and an increase in *Clostridium sensu stricto*.	[123]
Cao Q. et al.	2023	Summary statistics were obtained from MiBioGen and FinnGen and analysed using the MR-Egger, weighted median, inverse variance-weighted, and weighted mode methods.	*Allisonella* and *Bacteroides* are characterised by adverse effects on acne. *Ruminococcus torques* have a protective value against acne. *Candidatus soleaferrea*, *Eubacterium coprostanoligenes*, *Fusicatenibacter*, and *Lactobacillus* showed a suggestive association with acne.	[122]
Sivamani R.K. et al.	2023	Faecal samples from 17 patients with acne were analysed via shotgun whole-genome sequencing.	*Actinomyces naeslundii str Howell 279*, *Bifidobacterium dentium*, *Intestinibacter bartlettii DSM 16795*, and *Eubacterium sp AM28-29* had a positive correlation with the occurrence of non-inflammatory lesions. *Blautia obeum ATCC29174*, *Massilioclostridium coli*, *Schaalia odontolytica*, *Adlercreutzia equolifaciens subsp celatus*, and *Butyricicoccus sp GAM44* had a negative correlation with the occurrence of non-inflammatory lesions. *Coprococcus sp AF16-22*, *Butyrivibrio crossotus DSM 2876*, *Clostridium sp AF23-8*, *Escherichia coli KTE51*, *Akkermansia muciniphila ATCC BAA-835*, *Bilophila wadsworthia 316*, and *Methanobrevibacter smithii DSM2375* had a positive correlation with inflammatory lesions. *Coprococcus sp ART55-1* and *Alistipes senegalensis JC50* had a negative correlation with inflammatory lesions.	[124]

**Table 4:** Studies on the intestinal microflora in patients with alopecia areata.

Research	Year	Methodology	Key Results	References
Moreno-Arrones O.M. et al.	2020	Stool samples from 30 adult individuals (15 patients with alopecia areata and 15 controls) were analysed by 16S rRNA sequencing method.	No statistically significant differences in alpha and beta diversity between patients and controls. Patients affected by alopecia had a higher abundance of *Holdemania filiformis*, *Erysipelotrichacea*, *Lachnospiraceae*, *Parabacteroides johnsonii*, *Clostridiales vadin BB60 group*, *Bacteroides Eggerthii*, and *Parabacteroides distasonis*.	[141]
Lu J. et al.	2021	Analysis by 16S rRNA sequencing of stool samples from 33 AA patients and 35 control patients.	There were no statistically significant differences in alpha diversity between patients with AA and the control group. Three OTU biomarkers associated with AA were selected: OTU1237 (*Achromobacter*), OTU257 (*Megasphaera*), and OTU1784 (*Lachnospiraceae Incertae Sedis*).	[142]
Rangu S. et al.	2021	Analysing stool samples from 41 children with AA and 41 of their healthy siblings. Shotgun metagenomic sequencing.	There was a small but statistically significant difference in alpha and beta diversity. The relative abundance of *Ruminococcus bicirculans* was reduced in patients with alopecia areata compared to controls.	[143]
Brzychcy K. et al.	2022	Stool samples were collected from 25 adult patients with AA and examined by metataxonomic analysis of the full-length 16S V3-V4 sequencing.	The core microbiome of AA patients is formed by four main genera (*Lachnoclostridium*, *Bifidobacterium*, *Streptococcus* and *Eubacterium*) and three main types (*Firmicutes*, *Proteobacteria* and *Actinobacteria*). A loss of overall richness and a reduction in taxonomic diversity was observed in all samples.	[13]

## Data Availability

Not applicable.
